# Prevalence and Antimicrobial Resistance in *Salmonella* and *Shigella* Species Isolated from Outpatients, Jimma University Specialized Hospital, Southwest Ethiopia

**DOI:** 10.1155/2016/4210760

**Published:** 2016-08-23

**Authors:** Tesfahun Lamboro, Tsige Ketema, Ketema Bacha

**Affiliations:** Department of Biology, College of Natural Sciences, Jimma University, P.O. Box 378, 1000 Jimma, Ethiopia

## Abstract

This study was designed to investigate the prevalence of* Salmonella* and* Shigella* among outpatients in Jimma University Specialized Hospital, Southwest Ethiopia. Cross-sectional study was conducted involving a total of 176 outpatients. Stool specimens from both adult and pediatric outpatients were collected and analyzed for the presence of presumptive* Salmonella* and* Shigella* colonies followed by confirmation by biochemical tests. Pure cultures of* Salmonella* and* Shigella* species were further subjected to test for antibiotic resistance against the commonly used antibiotics. Furthermore, growth potential of the isolates in selected foods items was assessed following standard procedures. The result indicated that the prevalence of* Salmonella* and* Shigella* among outpatients in the study area was 19 (10.8%) and 2 (1.1%), respectively. In addition,* Salmonella* species were resistant to ampicillin (100%) followed by tetracycline (47.4%) and nalidixic acid (26.3%) while* Shigella* species were highly resistant to ampicillin and tetracycline (100%, each). Multidrug resistance towards maximum of four drugs was observed in both pathogens. The pathogens were observed growing to their infective dose within 24 hours. In conclusion,* Salmonella* and* Shigella* are still among microbes of public health importance in the study area. Thus, this calls for frequent monitory and evaluation of their prevalence and drug resistance patterns besides awareness development on water sanitation and hygienic food handling practices to the public at large.

## 1. Introduction

Infections associated with* Salmonella* and* Shigella* are among the major global public health problems. More than one billion cases of diarrhea occur worldwide due to nontyphoidal* Salmonella* every year leading to 3 million deaths [1]. Ninety-nine percent of the 200 million cases and more than 650,000 deaths per year due to infection with* Shigella* occur commonly in developing countries, primarily among children and young adults [2].* Salmonella* and* Shigella* cause mild to severe forms of intestinal tract infection commonly associated with consumption of a variety of foods [3].

*Salmonella* is a leading cause of foodborne illness worldwide and can cause enterocolitis (salmonellosis), enteric fever (typhoid fever), and septicemia with general symptoms of fever, diarrhea, abdominal cramps, nausea, vomiting, chills, and prostration. Usually the disease lasts a few days and is self-limited although occasionally the infection can be more serious, with loss of fluid and electrolytes, and can be fatal, especially to the sick, infants, and the elderly [1, 2, 4].

*Shigella* species are limited to the intestinal tract of humans and cause bacillary dysentery leading to watery or bloody diarrhea. Humans appear to be the only normal host reservoir for* Shigella* and they become infected by ingestion of contaminated food and water [5]. It is a highly infectious disease worldwide and its prevalence is the highest in tropical and subtropical regions of the world where living standard is very low and access to safe and adequate drinking water supply and proper excreta disposal system are often very limited or even absent [2, 5].

Ethiopia, as developing and tropical country, is frequently subjected to salmonellosis and shigellosis [6, 7]. There are several studies on prevalence of* Salmonella* or* Shigella* in Ethiopia, but they are restricted to health facilities in some age groups: mainly pediatric or adults [8–11]. To date, however, there was no report made on the prevalence of these pathogens in both children and adult outpatients. Thus, this study was designed to determine the prevalence of* Salmonella* and* Shigella* in representative outpatients in Jimma University Specialized Hospital (JUSH) and to evaluate drug resistance patterns among the isolates.

## 2. Materials and Methods

### 2.1. Description of the Study Area

The study was conducted in Jimma town, located 353 km southwest of Addis Ababa, the capital city of Ethiopia. The town is geographically located at 7°41′N latitude, 36°50′E longitude, and an average altitude of 1, 780 meters above sea level. Jimma town is generally characterized by warm weather with mean annual maximum and minimum temperature of 30°C and 14°C, respectively. The annual rainfall ranges between 1138 and 1690 millimeters [12]. Annually, Jimma University Specialized Hospital provides services for about 9000 inpatients and 80,000 outpatients, including clients of diverse socioeconomic and ethnic backgrounds, and bed capacity of 450 and a total of more than 550 staff members (https://www.ju.edu.et/jimma-university-specialized-hospital-jush).

### 2.2. Study Design and Population

Cross-sectional study design was employed involving outpatients of Jimma University Specialized Hospital. Diarrheal adult and pediatric outpatients who fulfilled the inclusion criteria (any outpatients visiting the hospital during the study period and had diarrhea) were enrolled in the study. Those outpatients who had taken antibiotic a week prior to data collection time were excluded from the study. Accordingly, a total of 176 outpatients who fulfilled the inclusion criteria were enrolled in the study.

### 2.3. Sample Collection and Microbial Analysis

One-gram stool sample was collected from each patient using sterile screw capped tubes containing transport media [9 mL buffered peptone water (Oxoid, UK)] and transported to Jimma University, Research and Postgraduate Laboratory for microbial analysis. After 24 hr incubation at 37°C, 1 mL of the sample was transferred into 10 mL selenite F broth (Oxoid, UK) and incubated. A loopful of culture was transferred onto xylose-lysine-deoxycholate agar (XLD) (Oxoid, UK) and incubated. The typical colonies were then further characterized based on colony morphology (*Shigella* appears as pink to red colonies on XLD, while* Salmonella* appears as red with black center). The cell morphology of pure culture was assessed by Gram staining. The morphological study includes cell shape, cell arrangement, presence or absence of endospore, and motility. Results of the isolates Gram reaction test were further confirmed with the rapid KOH method [13]. The isolates capability of catalase production, hence formation of bubbles, was checked using a 3% H_2_O_2_ solution.

For identification of* Shigella* and* Salmonella* spp., all suspected colonies were inoculated into appropriate biochemical media including Triple Sugar Iron Agar (TSIA), Lysine Iron Agar (LIA), Urea Agar (UA), Simmon's Citrate Agar (SCA), and Sulfide Indole Motility (SIM) medium.

### 2.4. Antimicrobial Sensitivity Test

Antimicrobial susceptibility of 19* Salmonella* spp. and 2* Shigella* spp. was carried out by disc diffusion method using Mueller-Hinton agar and nine commonly used commercial antibiotics. The standard antibiotic discs (and their potency) used in the current study included ampicillin (10 *μ*gmL^−1^), nalidixic acid (30 *μ*gmL^−1^), amikacin (30 *μ*gmL^−1^), tetracycline (30 *μ*gmL^−1^), chloramphenicol (30 *μ*gmL^−1^), norfloxacin (10 *μ*gmL^−1^), gentamycin (10 *μ*gmL^−1^), ciprofloxacin (5 *μ*gmL^−1^), and cotrimoxazole (25 *μ*gmL^−1^). A reference strain of* E*.* Coli* ATCC 25922 was used as quality control.

A standardized suspension of the bacterial isolate was prepared and turbidity of the inoculum was matched with 0.5 McFarland turbidity standard [14]. When bacterial culture containing the isolates matched the standard which was kept dark at room temperature, the culture was swabbed by cotton swab onto the Muller-Hinton Agar (Oxoid) and allowed to dry. Thereafter, the antibiotic discs were dispensed using sterile forceps on the medium and incubated at 37°C for 18 hr and the zones of inhibition were measured. The results of the antimicrobial susceptibility were interpreted based on the guidance of National Committee for Clinical Laboratory Standards [15] and the isolates were classified as sensitive, intermediate, or resistant. Intermediates were considered as resistant for purpose of analysis.

### 2.5. Growth Potential of* Salmonella* and* Shigella* Isolated from Diarrheal Outpatients on Selected Food

Growth potential of* Salmonella* and* Shigella* isolated from diarrheal outpatients was assessed on two local food items (gruel, which is made from meat, and firfir, made from cereal) frequently utilized by the community. Briefly, 200 g of each food item was steamed at 100°C, for a minute. Thereafter, 100 g of each food item was challenged with overnight culture of the isolates to give an inoculum level of 10^2^–10^3^ cfu/g. The challenged foods were left at ambient temperature for 24 hours. To investigate the initial inoculum level, inoculated foods (10 g each) were homogenised separately in 90 mL of buffered peptone water; and 0.1 mL of appropriate dilutions was spread-plated on XLD agar to count* Salmonella* and* Shigella*. Ten-gram portions of the food samples were further sampled aseptically at 6-hour intervals from 0 to 24 hours for microbial enumeration.

#### 2.5.1. Statistical Analysis

Data was analyzed using SPSS software version 16. All values were expressed as mean ± standard deviation and the mean values of counts of* Salmonella* and* Shigella* in the two food samples during challenge study were compared using one-way ANOVA. The significance of differences was considered at 95% confidence interval (*p* < 0.05).

### 2.6. Ethical Consideration

The study was ethically approved by Ethical Review Board of College of Natural Sciences. Written consent/assent for children <18 years old was obtained from guardians of the study participants prior to sample collection.

## 3. Results

### 3.1. Sociodemographic Characteristics of the Study Participants

A total of 176 diarrheal patients attending Outpatients Department of Jimma University Specialized Hospital were involved in the study with 100% response rate. About 33 (18.8%) of the outpatients were in the age category of <4 years (Table 1). The proportions of female outpatients (52.3%) were higher than males (47.7%) of which 63.6% were urban residents.

### 3.2. Prevalence of* Salmonella* and* Shigella*

A total of 19 (10.8%) and 2 (1.1%) outpatients among the 176 outpatients seeking medication at Jimma University Specialized Hospital were found positive for* Salmonella* and* Shigella*, respectively. The frequency of isolation of* Salmonella* was the highest among outpatients aged between 20 and 24 and 5 and 9 years with none detected among those aged above 40 years (Table 2). The only two* Shigella* isolates (1.1%) were encountered among children <4 years old. With the available few positive samples, the detection rate of* Salmonella* was relatively higher among male outpatients (10, 5.7%) with almost equal rate of detection in rural and urban residents (5.7% and 5.1%, resp.) although the very low prevalence of* Shigella* (1.1%) was encountered among illiterate rural farmers families aged <4 years (Table 2).

#### 3.2.1. Antimicrobial Susceptibility Pattern of* Salmonella* and* Shigella* spp

All the 19 isolates of* Salmonella* spp. were susceptible to ciprofloxacin and norfloxacin (100% each) followed by gentamycin (94.7%), chloramphenicol (94.7%), and amikacin (89.5%) (Table 3). However, the highest frequency of resistance was observed for ampicillin (100%) followed by tetracycline (47.4%) and nalidixic acid (26.3%) (Table 3). One isolate of* Salmonella* and* Shigella* species was found resistant to three similar antibiotics including chloramphenicol, gentamicin, and cotrimoxazole. Regarding* Shigella* spp., the two isolates were susceptible (100%) to ciprofloxacin, norfloxacin, and gentamycin although resistance was observed for ampicillin and tetracycline (Table 3).

In line with the literary definition of multidrug resistance (MDR), resistant to more than one antimicrobial agent, the MDR profile of* Salmonella* spp. indicated that 42.1% of the isolates were resistant to two antibiotics followed by three (26.3%) and four (21.0%) antibiotics (Table 4). The maximum number of antibiotics resisted by* Salmonella* spp. was four although the highest MDR (42.1%) was observed for combinations of two antibiotics: TE/AMP (resistance to tetracycline and ampicillin). Over all, resistance to two antibiotics dominated the resistance profile of* Salmonella* spp. The two* Shigella* isolates were resistant to four antibiotics of different patterns (TET/AMP/NAL/SXT and C/TET/AMP/AMK) (Table 4) (the codes are abbreviated below Table 4). Even with the current proposed definition of MDR, acquiring nonsusceptibility to at least one agent in three or more antimicrobial categories,* Salmonella* isolates displayed MDR as they were nonsensitive to many antibiotics that fall within more than five antimicrobial categories including Aminoglycoside (gentamicin), Fluoroquinolones (ciprofloxacin), Penicillins (ampicillin), Phenicols (chloramphenicol), and Tetracyclines (tetracycline) (Tables 3 and 4).

### 3.3. Growth Potential of* Salmonella* and* Shigella*

**Figure 1:**
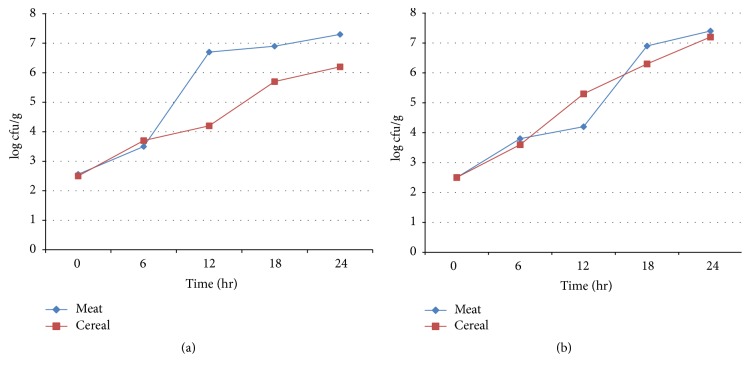
Growth potential of species of* Salmonella* (a) and* Shigella *(b) isolated from diarrheal outpatients on meat and cereal products (Jimma University Specialized Hospital, 2014).

Growth potential of* Salmonella* species was analyzed in gruel and firfir (meat and cereal based traditional foods, resp.) over a period of 24 hr. During the first 6 hr, nearly similar growth was observed in both food items (Figure 1(a)). Thereafter, the growth rate was increased by 3 log cfu/g in gruel (3.6–6.6 log cfu/g) with relatively slow growth rate (3.8–4.2 log cfu/g) in firfir until 12 hr. Finally, the count of* Salmonella* was as high as 7.2 log cfu/g and 6.2 log cfu/g in gruel and firfir, respectively, at the end of 24 hr storage.

Similarly, the growth potential of* Shigella* spp. was assessed in the same food items (gruel and firfir) as indicated for* Salmonella.* The growth rate was higher in the gruel sample (2.51–3.8 log cfu/g) than in firfir sample (2.5–3.5 log cfu/g) during the first 6 hr (Figure 1(b)). The growth rate increased by 1.5 log cfu/g (3.5–5.14 log cfu/g) in firfir within 12 hr and 3 log cfu/g (3.8–6.9 log cfu/g) in gruel within 18 hr. Maximum growth as revealed with counts of 7.5 log cfu/g (gruel) and 7.3 log cfu/g (firfir) was observed within 24 hr (Figure 1(b)).

**Figure 2:**
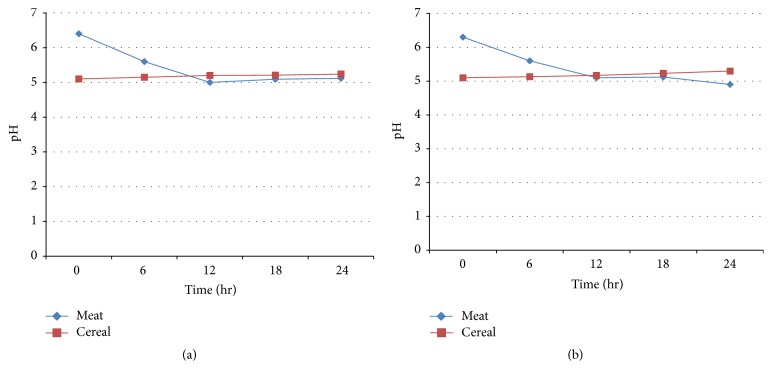
pH values of meat and cereal products challenged with species of* Salmonella* (a) and* Shigella* (b) isolated from diarrheal outpatients (Jimma University Specialized Hospital, 2014).

pH values of the challenged food samples varied over the period of 24 hr of storage. At the beginning (0 hr), the pH value of gruel (6.32) was greater than that of firfir (5.14). Thereafter, the pH value of gruel was reduced from 5.66 to 5.00 between 6 and 12 hr whereas that of firfir increased from 5.14 to 5.22. Finally, it slightly rose up and reached 5.1 for gruel and 5.28 for firfir (Figure 2(a)). Likewise, in* Shigella* challenged foods, at 0 hr of inoculation, the pH of gruel (6.32) was greater than the pH of firfir (5.14). Gradually, a slight pH reduction was observed in gruel up to 24 hr while the same was observed for firfir with only minor fluctuation (5.23 to 5.22) between 6 and 12 hr and it slightly increased and reached 5.28 at the end of 24 hr (Figure 2(b)).

## 4. Discussion

There are reports on widespread occurrence and distribution of* Salmonella* and* Shigella* in Ethiopia [8, 9, 11, 16, 17]. Recently, the number of* Salmonella and Shigella* related outbreaks in humans has still increased considerably in the same country [11]. Accurate estimates of the burden of diarrheal diseases caused by* Salmonella* species and other foodborne pathogens are needed to effectively set public health goals and allocate resources to reduce disease burden [18]. Accordingly, our finding indicated that, of the total 176 diarrheal outpatients, 10.8% were positive for* Salmonella* and 1.1% were positive for* Shigella*. The prevalence rate of* Salmonella* in this study is in agreement with that in the earlier studies reported as 10.7% [17], 11.5% [9], 13.6% [19], and 10.5% [11] but lower than that in a study reported as 15.4% [16] and higher than the 7.2% prevalence reported by Awole et al. [8].

In this study, the prevalence rate of* Shigella* was much lower than what was reported by Ashenafi and Gedebou [20] (9%) and Asrat et al. [21] (11.7%) from Tikur Anbessa, Ethio-Swedish Children's Hospital, and a report by Reda et al. [9] (6.7%) from Harar, Ethiopia. The low isolation rate of* Shigella* in this study is comparable with the very recent report (2.3%) made from among diarrheal children in Jimma Health Center [10]. The low prevalence of the target pathogens, especially* Shigella*, in the current study could be attributed to improved awareness of the community about personal and environmental hygiene from the continuous interventions being made by different stakeholders including the Health Science students of Jimma University through the educational program called Community Based Education.

Several studies showed possible differences in the frequency of isolation of* Salmonella* and* Shigella* infection among different age groups [11, 22]. Accordingly, the highest isolation rate of* Salmonella* was observed in the age group between 20 and 24 (26.3%) and 5 and 9 (26.3%) as supported by earlier reports made from Ethiopia by Mache [16] and Mengistu et al. [11]. To the contrary,* Shigella* species were encountered only in the age group below five. This is in agreement with reports from different parts of the world including Ethiopia [7, 23]. Therefore, shigellosis occurs worldwide but is most common among pediatric age group in underdeveloped tropical countries including Ethiopia. Community based data on shigellosis are incomplete but most hospital data suggested that the case-fatality rate is the highest among children less than 5 years old particularly if there is malnutrition. In epidemic shigellosis, the rate is as high as 3.9% in children under the age of 1 and 19.3% for infants less than 4 months of age. The case fatality declines with increase in age [18, 23]. Understanding the prevalence rate among different age groups is important to target intervention and preventive measures based on their age group.

In relation to educational status and frequency of isolation, this study indicated that there was high isolation rate of* Salmonella* and* Shigella* among the illiterates, with 36.8% and 100% isolation rates, respectively. This result is comparable with earlier study made by Aziz et al. [24]. Education is vital to create awareness in the community with regard to the mechanism of management of infectious diarrhea and control of other factors that lead to this disease. Poor environmental sanitation, malnutrition, inadequate water supply, poverty, and limited education are the major factors implicated in the occurrence, spread, and severity of diarrheal disease [25].

Due to selective pressure created by the use of antimicrobials in food processing animals, the risk of antimicrobial resistance among food borne pathogens has increased [26]. Mobile elements such as plasmids and transposons facilitate the rapid spread of antibiotic resistant genes among bacteria [27]. In addition, high rates of antibiotic resistant bacteria may possibly result from inappropriate or uncontrolled use of antibiotics. Therefore, it is necessary to pay attention to hygienic food handling practice as well as avoiding uncontrolled use of antibiotics [28]. An increase in the antimicrobial resistance in* Salmonella* and* Shigella* makes the treatment of infection more challenging. Therefore, epidemiological information and monitoring system are necessary to control* Salmonella* and* Shigella* infection in public health sectors.

In agreement with studies conducted by Beyene and Tasew [10],* Shigella* isolates were susceptible to ciprofloxacin, gentamicin, and norfloxacin. The resistance of* Shigella* spp. towards ampicillin and tetracycline is in agreement with studies conducted by Roma et al. [29] who reported high rate of resistance of* Shigella* spp. to ampicillin (93%), erythromycin (90%), tetracycline (90%), and cotrimoxazole (56%). Asrat [21] also reported high rate of resistance of* Shigella* species to tetracycline (97.3%) and ampicillin (78.7%).

The high level of antibiotic susceptibility of* Salmonella* to ciprofloxacin and norfloxacin is in agreement with earlier studies reported from Ethiopia [10, 11]. The resistance of* Salmonella* towards ampicillin (100%) and tetracycline (47.4%) was in agreement with report made by Beyene and Tasew [10] where most of the* Salmonella* isolates were resistant to ampicillin. In the current study, multidrug resistance towards four drugs was observed in* Salmonella* and* Shigella*.

The challenge studies revealed that* Salmonella* species reached the infective dose (5 log cfu/g) within 12 and 18 hr in gruel and firfir, respectively. The maximum count obtained was 7.2 log cfu/g in gruel and 6.2 log cfu/g in firfir. As compared to the previous study [30], the maximum count obtained in this study was relatively smaller. The reason for this difference can be the acidic nature of the food and the nature of the ingredients from which the food was prepared. For the cause of typhoid, an individual should have a minimum oral dose of 10^5^ *S*.* typhimurium* whereas at least 10^9^ *S*.* typhimurium* cells are required to cause symptoms of toxic infection. It takes 12–24 hr incubation after a person takes contaminated food containing sufficient number of* Salmonella* to manifest disease symptoms such as diarrhea, vomiting, and fever [31].

Likewise,* Shigella* species grow to the level of infective doses within 6 to 12 hr. The pathogen could initiate a successful infection at this cell number. The maximum growth observed in the current study was relatively lower as compared to studies reported by Ashenafi and Gedebou [20]. The reason for this discrepancy is the relatively acidic nature of gruel at the end of 24 hr. Even though the gruel is relatively acidic,* Shigella* manage to grow to the maximum of >7 log cfu/g within 24 hr period. This is because the pathogen can manage to grow in low pH food items [32]. Since this food item is frequently utilized by babies, care should be taken when handling the food; extension of the food before use should also be avoided. The maximum growth of* Shigella* species in firfir was almost similar with a very minor increment in gruel. The growth of* Shigella* species in firfir was steadier than that in gruel. The pathogens reach their infective dose within 6 to 12 hr. This is in agreement with studies reported by Ashenafi and Gedebou [20].

## 5. Conclusion

Findings of the current study revealed higher prevalence (10.8%, *N* = 176) of* Salmonella* species, dominantly among outpatients aged less than five years. The prevalence of* Shigella* (1.1%) was significant as compared to* Salmonella.* Furthermore, all* Salmonella* spp. were resistant to ampicillin although more than 90% of the isolates were susceptible to ciprofloxacin, norfloxacin, gentamycin, and chloramphenicol. Besides other factors, the potential health risks of the observed prevalences of* Salmonella* and* Shigella* were revealed by luxurious growth of both pathogens in the commonly used foods in the study area. This calls for designing of strategies for better awareness development among the community on hygienic food and water handling practices besides appropriate control measures. Thus, result of the present study will strengthen the knowledge in the field of epidemiology of* Salmonella* and* Shigella* to generate further trials which may help policy makers in planning interventions for the at risk population in the field of water sanitation and hygienic food handling practice. Furthermore, the observed drug resistance in* Salmonella* and* Shigella* can be used as an input by the health institutes for appropriate drug subscription.

## Figures and Tables

**Table 1:** Sociodemographic characteristics of the study participants (outpatients), Jimma University Specialized Hospital, 2014.

Characteristics	Category	Number of respondents	Percent (%)
Age	<10	54	30.7
	10–19	39	22.2
	20–29	34	19.3
	30–39	26	14.8
	>39	23	13.1

Education level	Illiterate	52	29.6
	Grades 1–4	18	10.2
	5–8	21	11.9
	9–12	27	15.3
	>12	58	33.0

Gender	Male	84	47.7
	Female	92	52.3

Residence	Urban	112	63.6
	Rural	64	36.4

Occupation	Unemployed	96	54.5
	Businessmen	28	15.9
	Farmer	26	14.8
	Civil servant	26	14.8

**Table 2:** Prevalence of *Salmonella* and *Shigella *against sociodemographic characteristics of the study participants, Jimma University Specialized Hospital, 2014.

Characteristics	Alternative	Frequency number (%)	*Salmonella* positive number (%)	*Shigella* positive number (%)
Gender	Male	84 (47.7)	10 (52.63)	1 (50)
	Female	92 (52.3)	9 (47.37)	1 (50)

Residence	Urban	112 (63.6)	9 (47.37)	0 (0.0)
	Rural	64 (36.4)	10 (52.63)	2 (100)

Age	<10	54 (30.68)	6 (31.58)	2 (100)
	11–19	39 (22.15)	3 (15.79)	0 (0)
	20–29	34 (19.32)	7 (36.84)	0 (0)
	30–39	26 (14.77)	3 (15.79)	0 (0)
	>39	23 (13.07)	0 (0)	0 (0)

Educational	Illiterate	52 (29.6)	7 (36.84)	2 (100)
	1–4	18 (10.2)	2 (10.52)	0 (0.0)
	5–8	21 (11.9)	5 (26.31)	0 (0.0)
	9–12	27 (15.3)	1 (5.26)	0 (0.0)
	>12	58 (33.0)	4 (21.05)	0 (0.0)

Occupation	Unemployed	96 (54.5)	14 (73.68)	0 (0.0)
	Businessmen	28 (15.9)	3 (15.79)	0 (0.0)
	Farmer	26 (14.8)	2 (10.52)	2 (100)
	Civil servant	26 (14.8)	0 (0.0)	0 (0.0)

**Table 3:** Antimicrobial susceptibility pattern of *Salmonella* and *Shigella* spp. isolated from diarrheal outpatients in Jimma University Specialized Hospital, Jan–Mar, 2014.

Antimicrobial agents	Disc potency (*μ*gmL^−1^)	*Salmonella* spp.			*Shigella* spp.		
		Resistant number (%)	Intermediate number (%)	Sensitive number (%)	Resistant number (%)	Intermediate number (%)	Sensitive number (%)
Amikacin	30	—	2 (10.5)	17 (89.5)	—	1 (50)	1 (50)
Ciprofloxacin	5	—	—	19 (100)	—	—	2 (100)
Chloramphenicol	30	1 (5.2)	—	18 (94.7)	—	1 (50)	1 (50)
Gentamycin	10	1 (5.2)	—	18 (94.7)	—	—	2 (100)
Cotrimoxazole	25	1 (5.2)	5 (26.3)	13 (68.4)	1 (50)	—	1 (50)
Norfloxacin	30	—	—	19 (100)	—	—	2 (100)
Nalidixic acid	30	5 (26.3)	4 (21.05)	10 (52.6)	1 (50)	—	1 (50)
Ampicillin	10	19 (100)	—	—	2 (100)	—	—
Tetracycline	30	9 (47.4)	3 (15.7)	7 (36.8)	2 (100)	—	—

**Table 4:** MDR of *Salmonella *spp. and *Shigella* spp. isolated from diarrheal outpatients in Jimma University Specialized Hospital, Jan–Mar, 2014.

Number of antimicrobial resistance values	*Salmonella *spp.			*Shigella* spp.	
	Antimicrobial resistance patterns	Number of isolates (%)	Total (%)	Antimicrobial resistance patterns	Number of isolates
Two	TET/AMP	5 (26.3)	8 (42.1)	—	
	SXT/AMP	1 (5.2)		—	
	NAL/AMP	2 (10.52)		—	

Three	TET/NAL/AMP	2 (10.52)	5 (26.3)	—	
	SXT/AMP/TET	2 (10.52)		—	
	NAL/SXT/AMP	1 (5.2)		—	

Four	NAL/AMP/TET/SXT	1 (5.2)	4 (21.0)	TET/AMP/NAL/SXT	1
	NAL/TET/AMP/C	1 (5.2)		C/TET/AMP/AMK	1
	AMP/SXT/CN/NAL	1 (5.2)			
	AMK/AMP/NAL/TET	1 (5.2)			

TET: tetracycline, AMP: ampicillin, SXT: cotrimoxazole, NAL: nalidixic acid, C: chloramphenicol, CN: gentamycin, AMK: amikacin, CIP: ciprofloxacin, and NOR: norfloxacin.

## Abbreviations

XLD: Xylose lysine deoxycholate
KOH: Potassium hydroxide
H_2_O_2_: Hydrogen peroxide
TSIA: Triple Sugar Iron Agar
LIA: Lysine Iron Agar
UA: Urea Agar
SCA: Simmon's Citrate Agar
SIM: Sulfide Indole Motility
ATCC: American type culture collection
TET: Tetracycline
AMP: Ampicillin
SXT: Cotrimoxazole
NAL: Nalidixic acid
C: Chloramphenicol
CN: Gentamycin
AMK: Amikacin
CIP: Ciprofloxacin
NOR: Norflaxacin.
